# *Pseudomonas*-Specific NGS Assay Provides Insight Into Abundance and Dynamics of *Pseudomonas* Species Including *P. aeruginosa* in a Cooling Tower

**DOI:** 10.3389/fmicb.2018.01958

**Published:** 2018-08-21

**Authors:** Rui P. A. Pereira, Jörg Peplies, Douglas Mushi, Ingrid Brettar, Manfred G. Höfle

**Affiliations:** ^1^Department of Vaccinology and Applied Microbiology, Helmholtz Centre for Infection Research, Braunschweig, Germany; ^2^Ribocon GmbH, Bremen, Germany; ^3^Department of Biological Sciences, Sokoine University of Agriculture, Morogoro, Tanzania

**Keywords:** *Pseudomonas*, *Pseudomonas aeruginosa*, pathogens, freshwater, cooling tower, next generation sequencing, diagnostics

## Abstract

*Pseudomonas* species are frequent inhabitants of freshwater environments and colonizers of water supply networks via bioadhesion and biofilm formation. *P. aeruginosa* is the species most commonly associated with human disease, causing a wide variety of infections with links to its presence in freshwater systems. Though several other *Pseudomonas* species are of ecological and public health importance, little knowledge exists regarding environmental abundances of these species. In the present study, an Illumina-based next-generation sequencing (NGS) approach using *Pseudomonas*-specific primers targeting the 16S rRNA gene was evaluated and applied to a set of freshwater samples from different environments including a cooling tower sampled monthly during 2 years. Our approach showed high *in situ* specificity and accuracy. NGS read counts revealed a precise quantification of *P. aeruginosa* and a good correlation with the absolute number of *Pseudomonas* genome copies in a validated genus-specific qPCR assay, demonstrating the ability of the NGS approach to determine both relative and absolute abundances of *Pseudomonas* species and *P. aeruginosa*. The characterization of *Pseudomonas* communities in cooling tower water allowed us to identify 43 phylotypes, with *P. aeruginosa* being the most abundant. A shift existed within each year from a community dominated by phylotypes belonging to *P. fluorescens* and *P. oleovorans* phylogenetic groups to a community where *P. aeruginosa* was highly abundant. Co-occurrence was observed between *P. aeruginosa* and other phylotypes of *P. aeruginosa* group as well as the potentially pathogenic species *P. stutzeri*, but not with phylotypes of the *P. fluorescens* group, indicating the need to further investigate the metabolic networks and ecological traits of *Pseudomonas* species. This study demonstrates the potential of deep sequencing as a valuable tool in environmental diagnostics and surveillance of health-related pathogens in freshwater environments.

## Introduction

The genus *Pseudomonas*, currently comprising about 150 species, contains several species of high ecological importance that are linked to a large array of catabolic functions, i.e., bioremediation, biodegradation, and biosorption (Sayler et al., 1990; Banat, 1995; Zhang et al., 2005; Wasi et al., 2013). *Pseudomonas* have also been linked to biocorrosion due to adhesion and biofilm formation in freshwater infrastructures, such as cooling towers and drinking water supplies (Garrett et al., 2008; Liu et al., 2009; Dang and Lovell, 2016). Additionally, several *Pseudomonas* species are human pathogens, with *P. aeruginosa* as the most clinically relevant (Mena and Gerba, 2009). *P. aeruginosa* is a major pathogen in a wide variety of hospital-acquired infections and the main causative agent of respiratory tract infections in patients with bronchiectasis and cystic fibrosis, causing high health-care costs, clinical morbidity and mortality (Jones et al., 2002; Lyczak et al., 2002; Al-Aloul et al., 2004; Moradali et al., 2017). The clinical diseases associated to *P. aeruginosa* infections vary from mild to severe, e.g., skin lesions, tracheobronchitis, necrotizing bronchopneumonia, urinary tract infections, endocarditis, bacteremia, and meningitis (Lyczak et al., 2000; Rossolini and Mantengoli, 2005).

Freshwater has been identified as the main source of *P. aeruginosa* nosocomial infections, with strong links described between environmental and clinical isolates (Squier et al., 2000; Anaissie et al., 2002; Trautmann et al., 2005; Hota et al., 2009). The exposure of personnel and patients in hospital facilities to *P. aeruginosa* occurs by means of bacterial colonization, with biofilm formation in the plumbing system and transmission occurring initially through contact and aerosol generation in contaminated areas, and posteriorly by horizontal acquisition (Morrison and Wenzel, 1984; Döring et al., 1991; Bergmans et al., 1998; Trautmann et al., 2005). In addition, *P. aeruginosa* infections also occur frequently in healthy individuals, with outbreaks being mainly the result of contact with *P. aeruginosa* contaminated freshwater (Mena and Gerba, 2009). Besides, less frequent modes of transmission such as drinking water consumption and aerosol breathing have been described (Mena and Gerba, 2009).

Taking together, the monitoring of freshwater systems with the detection, quantification and qualitative description of *Pseudomonas*, and particular focus on the potentially pathogenic species, is of major relevance. Though *Pseudomonas* can easily grow and be recovered by traditional cultivation methods and a high number of species have been discovered, cultivation-dependent methods appear to underestimate *Pseudomonas* diversity and concentration when compared with molecular assays, supposedly due to their ability to transit into a viable but non-culturable state (VBNC) (Tarnawski et al., 2003; Lloyd-Jones et al., 2005; Oliver, 2010; Li et al., 2013). Several molecular assays, targeting the 16S rRNA gene sequence, have been developed to allow the detection and quantification of *Pseudomonas* species as well as the characterization of their diversity and population structure (Widmer et al., 1998; Johnsen et al., 1999; Spilker et al., 2004; Bergmark et al., 2012). Yet, to our knowledge, only one study applying 454 pyrosequencing has been directly applied to environmental samples, more precisely soil (Bergmark et al., 2012).

The increasing application of next-generation sequencing (NGS) in aquatic environments has provided us with insight into the microbial communities in freshwater systems, increasing our knowledge and understanding of abiotic and biotic factors that affect water quality and safety (Tan et al., 2015). Moreover, of the NGS platforms available, Illumina MiSeq has shown better performances compared to other platforms, such as 454 pyrosequencing, allowing a deeper and more accurate characterization and detection of bacterial taxa due to its highest throughput per run and lowest error rates (Loman et al., 2012). Nevertheless, to the best of our knowledge, no molecular studies based on a genus-specific Illumina approach have been conducted to evaluate the potential of NGS-based methods as a tool for the detection and quantification of *Pseudomonas* species, including *P. aeruginosa*, in freshwater environments.

In this present study, we aimed to develop a robust NGS assay to be applied for surveillance and monitoring of *Pseudomonas* species, including the most potentially pathogenic species, *P. aeruginosa*, in freshwater environments. In addition, this approach was applied to a set of cooling tower water samples collected over 2 years in order to determine the *Pseudomonas* community composition and its temporal dynamics.

## Materials and Methods

### Sites and Sampling Description

Drinking water was collected from the laboratory D.004 at the Helmholtz Centre for Infection Research (HZI) campus (Braunschweig, Germany). A description of the drinking water supply system is given in preceding publications (Eichler et al., 2006; Henne et al., 2013).

River water was sampled in October 2012 (dry season) from the Morogoro river, which belongs to the upper Ngerengere watershed in Tanzania at an altitude range of 500–2260 m above sea level. Water samples were taken from sampling points near distinct adjoining watershed land-use types [pristine forest (-6.8907, 37.5947); urban site (-6.8230, 37.6814); agricultural site (-6.7652, 37.7484)].

Cooling tower water was sampled monthly (2nd week), during the period January 2013 through December 2014, from a cooling tower in the HZI campus. The studied open-circuit cooling tower system is used for the discharge of waste heat generated by air conditioning and other heat producing processes at the campus. The make-up water consists of regular drinking water, while conductivity controls for water blowdown. Briefly, when exceeding 400 μS cm^-1^, a portion of the circulating water is discharged and replaced by drinking water until a conductivity of 350 μS cm^-1^ is reached. Disinfection of the system consisted of silver plus hydrogen peroxide on a continuous basis. Chlorine dioxide treatments were occasionally performed in 2014 (May 15th, August 12th, and October 15th). A thorough description of the system is given elsewhere (Pereira et al., 2017b). The measured physicochemical parameters of the water in the cooling tower system as well as the operational data concerning the system are listed on the **Supplementary Tables 1, 2**.

### Total Cell Counts of Cooling Tower Water Bacteria

To determine total bacterial cell counts (TCC), formaldehyde-fixed (2% final concentration) cooling tower water samples were stained with Sybr Green I dye (1:10000 final dilution, Molecular Probes, Invitrogen, Carlsbad, CA, United States) and analyzed directly by epifluorescence microscopy. The TCC were performed by counting 10 microscope fields (100-fold magnification) and images were analyzed by Image J software.

### Water Filtration and DNA Extraction

Drinking water and cooling tower water microorganisms were sampled by filtration according to a previously applied protocol (Eichler et al., 2006). Briefly, 3 and 5 liters of cooling tower water and drinking water, respectively, were filtered through a filter sandwich consisting of a 0.2 μm pore size polycarbonate filter (90 mm diameter, Nucleopore, Whatman, Maidstone, United Kingdom) with a precombusted glass fiber filter on top (90 mm diameter; GF/F; Whatman, Maidstone, United Kingdom). The filters were stored at -70°C until DNA extraction using a modified DNeasy Blood & Tissue protocol (Qiagen, Hilden, Germany) described in a preceding publication (Henne et al., 2013).

Two liters of river water were sampled aseptically from the midstream of the Morogoro River, at a depth of approximately 30 cm, using 1 liter wide-mouthed sterile plastic bottles (Thermo Scientific Nalgene, Neubrecht, Lima, OH, United States). The two liters of river water were then filtered onto a 0.2 μm-pore-size polycarbonate filter (47 mm diameter, Nucleopore, Whatman, Maidstone, United Kingdom). Bacteria were recovered by scraping the filter surface, resuspended in Milli-Q water (Merck Millipore, Darmstadt, Germany) and stored on FTA cards (GE Healthcare UK Limited, Buckinghamshire, United Kingdom). A total of 6 FTA punches (6 mm diameter) were made, and environmental genomic DNA was extracted using the UltraClean PowerWater DNA isolation kit (Mo Bio laboratories, Carlsbad, CA, United States), following the manufacturer’s instructions.

### Design of *Pseudomonas* NGS Libraries

The primers used for the construction of the *Pseudomonas* NGS libraries were designed to include the 16S rRNA gene complementary regions plus the complementary sequences to the Illumina specific adapters and the flow cell binding sites of the Illumina MiSeq platform. The amplification of the 16S rRNA gene V3 and V4 regions of genus *Pseudomonas* was achieved using the primer pair Pse434F (5′-ACTTTAAGTTGGGAGGAAGGG-3′) and Pse665R (5′-ACACAGGAAATTCCACCACCC-3′) (Bergmark et al., 2012). The NGS libraries construction via a two-step PCR-based amplification with the *Pseudomonas*-specific customized primers was performed using a dual-indexing approach previously published (Pereira et al., 2017a). Briefly, the forward primer included a six-nucleotide index (barcode), of a total of 20 sample-specific indices selected from the error-correcting indices listed previously (Bartram et al., 2011). The barcodes selected respected a minimum Hamming distance of 3 and had a two-nucleotide linker (“CA”). The reverse primer integrated an unique index selected from twelve used in this study, which are detailed in Illumina library preparation protocols, in order to allow multiplexing of samples. The detailed primer sequences of the first and second PCR are listed on the **Supplementary Table 3**. All primers were synthesized and HPSF purified by Eurofins MWG Operon (Ebersberg, Germany).

After the second PCR amplification, the libraries were size-selected by gel electrophoresis on a 2% agarose gel pre-stained with GelRed Nucleic Acid Gel Stain (Biotium, Hayward, CA, United States), and recovered using a slightly modified QIAquick Gel Extraction Kit (Qiagen, Hilden, Germany) protocol (Pereira et al., 2017a). The DNA concentration of the extracted amplicons was quantified by Quant-iT Picogreen dsDNA assay kit (Life Technologies, Oregon, United States) on a VICTOR *X*3 2030 Multilabel Plate Reader (Perkin Elmer, Germany) to allow, when required, equimolar pooling. Subsequently, pooled libraries were purified by the MinElute PCR Purification Kit (Qiagen, Hilden, Germany). Molarity was quantified and library fragment size confirmed with Agilent Bioanalyzer. The sequencing of the libraries was completed by the Genome Analysis Department of the HZI, using the Illumina MiSeq platform (V2 chemistry, 250 bp paired-end run).

### Amplification of *Pseudomonas*-Specific 16S rRNA Gene

The *Pseudomonas*-specific first-step PCR reaction mixture (50 μL) consisted of 0.1 mM of each dNTP (Bioline, Luckenwalde, Germany), 1.5 mM MgCl_2_, 1× PCR reaction buffer, 0.03 U of HotStarTaq Polymerase (Qiagen, Hilden, Germany), 0.4 μM of each primer and 5 ng of environmental DNA. Cycling was performed on a Biorad thermal cycler 96-well iCycler. The PCR included an initial denaturation for 15 min at 95°C; 30 cycles of 1 min at 95°C, 30 s at 58°C, 30 s at 72°C followed by a final extension at 72°C for 10 min. 2 μL of the PCR product was used as a template for a second PCR. The 50 μL mix consisted of 0.1 mM of each dNTP (Bioline, Luckenwalde, Germany), 0.75 mM MgCl_2_, 1× PCR reaction buffer, 0.03 U of HotStarTaq Polymerase (Qiagen, Hilden, Germany) and 0.4 μM of each primer. The PCR conditions involved a cycle at 95°C for 15 min, 10 cycles of 95°C for 45 s, 57°C for 30 s, and 72°C for 30 s, ending with an extension at 72°C for 10 min.

### Accuracy, Sensitivity, and Quantitative Precision of NGS Approach

To determine the sequence accuracy (error rate) and quality (error-free sequences) of the sequenced libraries using the developed assay, internal controls (triplicates) consisting of nuclease-free water samples spiked with genomic DNA of *P. aeruginosa* DSM 50071^T^ (GPS-Genetic PCR Solutions, Alicante, Spain) were added to the same NGS run. 5,000 paired-end merged reads of each sample were statistically sampled and an alignment algorithm was applied against the expected *P. aeruginosa* reference 16S rRNA sequence.

To assess the sensitivity and quantitative precision (agreement between the read abundance observed and the phylotype concentration in the sample) of the NGS approach, ten-fold dilutions (range: 10^1^–10^5^) of a certified genomic standard of *P. aeruginosa* DSM 50071^T^ (GPS-Genetic PCR Solutions, Alicante, Spain) were separately added to triplicate environmental DNA aliquots (5 ng) of two *P. aeruginosa*-negative freshwater samples (cooling tower and drinking water). For comparison, these samples were also analyzed in the same run without spiking of *P. aeruginosa*. The genomic standard was also spiked into nuclease-free water for evaluation of inhibition by the environmental matrix in the environmental water samples.

### Absolute Quantification of *Pseudomonas* Species by NGS

For the NGS-based absolute quantification of *Pseudomonas* species in cooling tower water samples, ten-fold dilutions (range: 10^1^–10^6^) of a certified genomic standard of *P. aeruginosa* DSM 50071^T^ (GPS-Genetic PCR Solutions, Alicante, Spain) were prepared and spiked to nuclease free water to achieve a total template volume of 5 μL. These samples were processed, in triplicate, by the *Pseudomonas*-specific NGS assay to generate the libraries.

In addition, environmental genomic DNA (5 ng in 5 μL) from twelve cooling tower samples, monthly sampled in 2014, were simultaneously amplified, in triplicate, by the *Pseudomonas*-specific NGS assay to generate the libraries. The NGS libraries of the samples containing nuclease-free water spiked with genomic DNA of *P. aeruginosa* were pooled (equivolume pooling) together with the NGS libraries of the twelve cooling tower samples and run in the Illumina MiSeq platform.

For the absolute quantitative determination of *Pseudomonas* species based on the NGS 16S rRNA reads, a standard curve was generated and the resulting equation used to estimate the *Pseudomonas* genome copies from the quantity of *Pseudomonas* reads generated for the cooling tower water samples. The *Pseudomonas* genome copies per liter of cooling tower water were calculated as the product of the *Pseudomonas* genome copies measured by NGS in 1 ng of environmental DNA extracted and the total amount of DNA extracted in 1 liter of cooling tower water filtered.

### *Pseudomonas*-Specific qPCR Assay and Quantification of Total Bacteria by Real-Time PCR

The optimized qPCR consisted of a total volume of 25 μL reaction mixture volume containing 5 ng of environmental DNA, 10 μL of 2× SYBR Green Master Mix (Roche Diagnostics, Mannheim, Germany) and 125 nM of each primer (Pse434F and Pse665R). The qPCR assay was performed on a LightCycler 480 system (Roche Diagnostics, Mannheim, Germany). Amplification cycling comprised an initial cycle at 95°C for 5 min, followed by 40 cycles of denaturation at 95°C for 20°s, annealing at 60°C for 20 s and extension at 72°C for 25 s. Then melting curves were determined using SYBR Green fluorescence with one cycle of the following program: 10 s at 95°C, followed by 60 s at 65°C and a final continuous reading step of 7 acquisitions per second between 65°C and 98°C.

The absolute number of *Pseudomonas* cells (Psecounts) was determined by calculating the ratio between *Pseudomonas* spp. 16S rRNA gene copies and total bacterial 16S rRNA gene copies, while considering a mean of 4.2 16S rRNA gene copies per genome for the bacterial community (Větrovský and Baldrian, 2013) and of 4.8 for *Pseudomonas*^1^. This ratio was subsequently multiplied by the total cell counts (TCC) determined by the epifluorescence microscopy. The formula used to calculate the absolute number of *Pseudomonas* cells (Pse_counts_) is the following:

$${\text{Pse}}_{\text{counts}}\;=\;\frac{\text{Pse16S/4}.\text{8}}{\text{Bact16S/4}.\text{2}}\;\times \;\mathrm{TCC}$$

The *Pseudomonas* 16S rRNA gene copies per liter of cooling tower water (Pse16S) were calculated as the product of the *Pseudomonas* 16S rRNA gene copies measured by qPCR in 1 ng of environmental DNA extracted and the total amount of DNA extracted in 1 liter of cooling tower water filtered. The total bacterial 16S rRNA gene copies per liter of cooling tower water (Bact16S) were calculated as the product of the bacterial 16S rRNA gene copies measured by qPCR in 1 ng of environmental DNA extracted and the total amount of DNA extracted in 1 liter of cooling tower water filtered. The total bacterial 16S rRNA gene abundance was quantified according to an existing qPCR assay as previously described (Lesnik et al., 2016).

### NGS Data Quality Filtering and Taxonomic Assignment

Pre-processing of Illumina MiSeq reads was done using mothur (v.1.34.0) (Schloss et al., 2009). Quality control check, alignment of reads and classification to genus-level were executed using the SILVA pipeline as previously described (Pruesse et al., 2007; Pereira et al., 2017b). Filtered reads were dereplicated and clustered with cd-hit-est (Li and Godzik, 2006) using identity criterion level of 1.00 and 0.99, respectively. OTUs with less than 5 reads were removed. For species-level classification, NGS sequences after trimming of primer sequence were blasted using BLAST+ (version 2.2.30) (Camacho et al., 2009) against a database including curated and truncated 16S rRNA sequences of *Pseudomonas* species that were retrieved from the SILVA SSU 115 NR dataset (Quast et al., 2013). Sequences were assigned to a species when sequence identity was > 98%. This threshold was defined after assessment of the sequence accuracy of the *P. aeruginosa* NGS libraries generated by the assay. When the V3-V4 16S rRNA gene fragments of previously described isolates of distinct *Pseudomonas* species showed ≤1 mismatch, sequences were assigned to species clusters (**Supplementary Figure 1**). Species and species clusters will be throughout the study equally designated as phylotypes. Grouping of phylotypes to phylogenetic groups was achieved according to DNA-sequence based analyses of the genus *Pseudomonas* (Mulet et al., 2010; Gomila et al., 2015).

**FIGURE 1:**
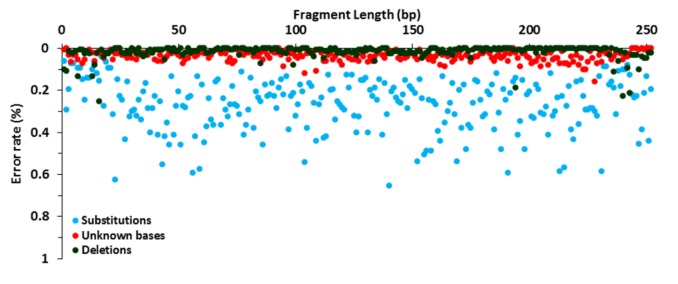
Error rate distribution. Analysis of the NGS library preparation, Illumina MiSeq amplification and sequencing using 15,000 reads of *P. aeruginosa* DSM 50071^T^. Substitution (blue), unknown base (red), and deletion (green) rates are shown.

### NGS Data Archive

The raw partial 16S rRNA gene sequence data obtained from the application of the *Pseudomonas*-specific approach are stored in the European Nucleotide Archive (ENA) under the accession number PRJEB14849.

### NGS Data Analysis

Alpha-diversity metrics were obtained by Explicet (Robertson et al., 2013). Multivariate analyses were performed using PRIMER (Version 7.0.7) (Clarke and Warwick, 2014). For evaluation of repeatability (within-NGS run precision) of technical replicates, weighted Spearman rank correlation was calculated and visualized using group-average hierarchical clustering. Sample cluster analysis was done with SIMPROF (Clarke, 1993). For inter-sample comparison of *Pseudomonas* communities, Bray–Curtis similarity matrices were generated, by comparing the standardized, untransformed abundances of taxa. Data are represented by non-metric multidimensional scaling (nMDS) plots. Potentially significant differences between groups of samples were analyzed using ANOSIM (Clarke, 1993), with a statistically significant difference considered if *P*-value < 0.05. Index of association (Type 3 SIMPROF) (Somerfield and Clarke, 2013) and Spearman correlations were calculated to evaluate phylotypes temporal associations. Correlation of abiotic and biotic data was assessed using distLM in PRIMER (Version 7.0.7) add-on PERMANOVA+.

## Results

### Sequence Accuracy of *Pseudomonas* Libraries

The raw error rate of the library amplification and sequencing steps was determined using *P. aeruginosa* DSM 50071^T^ as a template. Substitution, indel and unknown base (Ns) rates were calculated after alignment to a reference sequence and their distribution along the 16S rRNA gene amplicons was characterized. The analysis of 3,780,000 bases, corresponding to 252 bases in 15,000 full-length reads processed, showed a low global error rate with a mean value of 0.318% ± 0.13%. The minimum and maximum error rate per base were 0.073% and 0.701%, respectively. Erroneous sequencing was detected in all base positions along the full-length fragment in one or more reads. When breaking down the global error rate by error type, substitutions were found to be the most common errors (0.266%), followed by unknown bases (0.037%), deletions (0.012%), and insertions (0.003%) (**Supplementary Figure 2A**). Of the total 10,067 substitutions, 7765 (77.1%) were transitions while only 2,302 (22.9%) were transversions (**Supplementary Figure 2B**).

**FIGURE 2:**
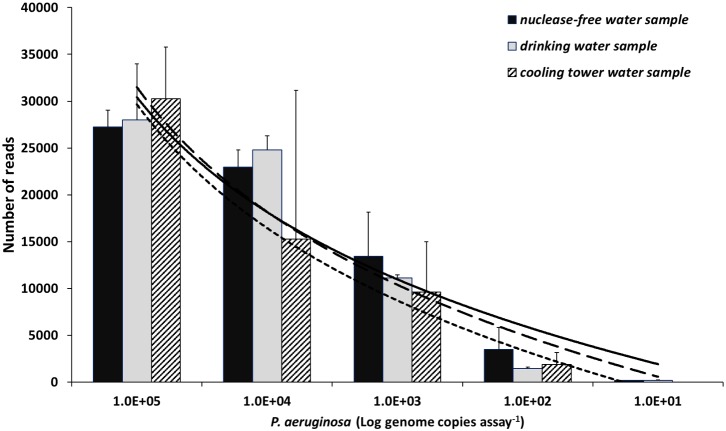
Read abundance relationship with concentration values of *P. aeruginosa*. Data generated after spike-in of three samples: nuclease-free water (black), drinking water (gray), and cooling tower water (hatched). A logarithmic regression model explains the relationship between the two variables. Solid, dashed, and square-dot logarithmic curve show correlation for nuclease-free water, drinking water, and cooling tower water samples, respectively. The coefficient determination (*R*^2^) values were 0.96, 0.94, and 0.92 for the spiked nuclease-free water, drinking water, and cooling tower water samples, correspondingly. Y-axis represents mean number of reads of replicates (*n* = 3). Data presented as mean + SD. Note the x-axis in log scale.

The comparison of error presence, distribution and variation along the sequence showed different behavior according to the type of error (**Figure 1**). Substitutions were found at all positions while unknown bases and deletions were detected in 95.6 and 66.7% of the 252 bases, respectively. Moreover, indel distribution was strongly uneven, with 31.6 and 36.6% of the total indels presented in the sequenced fragments corresponding to the forward and reverse primer sequences, respectively. Overall, 54.1% of the reads were error-free, with 99.6% of the reads showing a sequence similarity higher than 98% to *P. aeruginosa*. By using this threshold, only 0.03% of the reads were misclassified. As a result of the high accuracy of the generated libraries, only the sequence fragments complementary to the primer sequences were removed prior to taxonomic assignment and a similarity threshold of 98% was used for species classification in this study.

### *In situ* Evaluation of *Pseudomonas*-Specific Primers and Repeatability of NGS Assay

For the *in situ* analysis of primer specificity and evaluation of the repeatability of the library preparation protocol, triplicates of nine freshwater samples from three different freshwater environments [four cooling tower water samples (1–4), four river water samples (5–8) and one drinking water sample (9)] were barcoded and sequenced on the Illumina MiSeq platform. A total of 731, 971 V3–V4 classified reads were processed, with a mean number of 27,110 ± 15,778 reads per sample, after quality filtering. When analyzing the reads, in respect to the taxonomic groups these were classified to, 721,649 (98.6%) were affiliated to *Pseudomonas*. Twenty-four non-*Pseudomonas* taxa were detected in the studied freshwater samples with the most abundant genera belonging to class *Gammaproteobacteria* and included in the orders *Legionellales* and *Pseudomonadales*. *Acinetobacter* was the most representative (1.26 ± 1.83%) genus, followed by *Aquicella* (0.25 ± 0.41%), *Azomonas* (0.23 ± 0.43%) and *Azotobacter* (0.16 ± 0.33%). The *in situ* results are mostly in accordance to the predictive *in silico* analysis of the primer pair amplification of taxa closely related to *Pseudomonas*, i.e., *Azotobacter* (95% coverage), *Azomonas* (89%), and *Acinetobacter* (8%).

To evaluate repeatability, we removed OTUs with read number lower than 5, which lead to a reduction of processed reads to 695, 888. The observation of OTU numbers leveling off at the rarefaction curves (**Supplementary Figure 3**), together with a high Good’s coverage value (98.6 ± 0.3%), indicate adequate sequencing effort. When carrying out a phylotype hierarchical clustering based on weighted Spearman rank correlation, a separation in two main clusters was clearly observed; one including the river water samples (5 to 8), and the other both cooling tower and drinking water samples (1 to 4 and 9) (**Supplementary Figure 4**). Moreover, while significant differences were detected between samples (ANOSIM, *R* = 0.95, *P* < 0.05), no significant community structure dissimilarities were observed between replicates within any of the freshwater samples (ANOSIM, *P* > 0.05). Though results showed an overall weighted Spearman rank correlation coefficient mean value of 0.80, values varied substantially between the studied samples, ranging from 0.72 ± 0.08 to 0.97 ± 0.01 (**Supplementary Table 4**). This rank-based variation was correlated to the absolute concentration of *Pseudomonas* present in the sample (*r_s_* = 0.71, *P* < 0.05). A Bray–Curtis similarity abundance-based analysis revealed identical results with a mean index value among replicates of 81.8 ± 8.9%. The similarity metrics among replicates negatively correlated with the *Pseudomonas* community diversity (*r_s_* = -0.7, *P* < 0.05).

**FIGURE 3:**
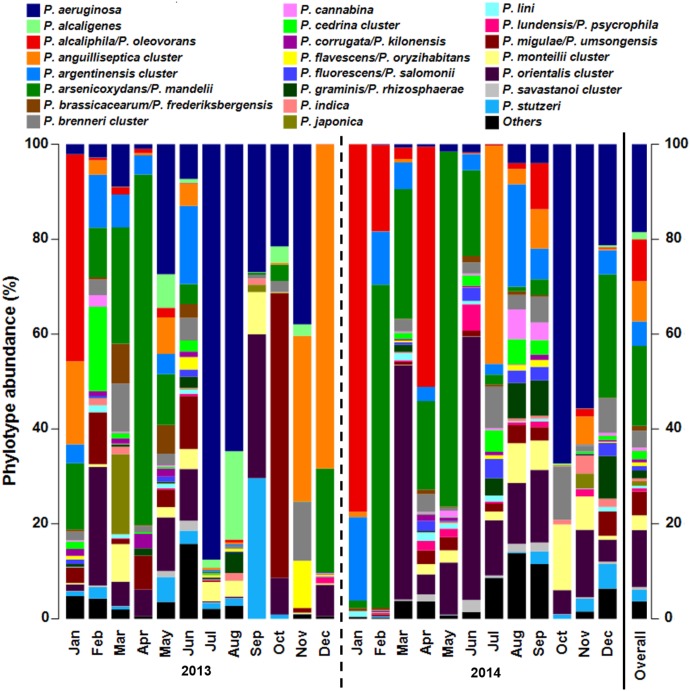
*Pseudomonas* community composition of cooling tower water samples assessed by genus-specific 16S rRNA gene amplicon sequencing with Illumina MiSeq. Data presented as bar chart showing relative abundance of phylotypes (%). Phylotypes can correspond to species or a cluster of species according to amplified fragment resolution. The species present in each cluster are described in **Supplementary Figure 1**. Bar chart of data analyzed according to *Pseudomonas* phylogenetic species groups is shown in **Supplementary Figure 6**. Samples collected monthly in 2013 and 2014 are separated by year with a dashed line. Bar designated as Overall presents mean relative abundance of *Pseudomonas* phylotypes for all samples studied. Jan, January; Feb, February; Mar, March; Apr, April; Jun, June; Jul, July; Oct, October; Nov, November; Dec, December.

**FIGURE 4:**
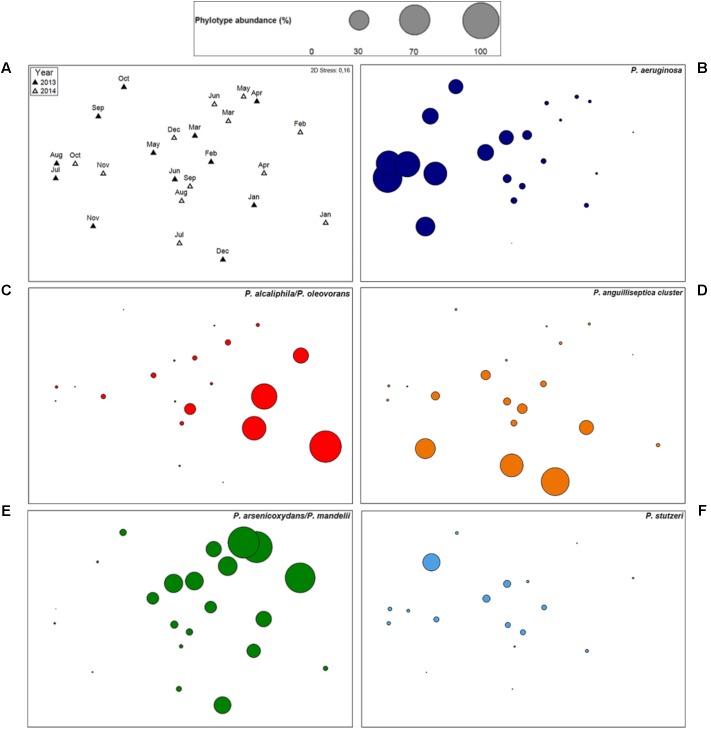
*Pseudomonas* community structure and dynamics of individual phylotypes. Non-metric multidimensional scaling (nMDS) plot **(A)** comparing the *Pseudomonas* community structure of cooling tower water samples, sampled from January 2013 to December 2014. Phylotypes standardized untransformed data was used for calculation of Bray–Curtis similarity. Filled and empty triangles represent 2013 samples and 2014 samples, respectively. Kruskal 2D Stress: 0.16. **(B–F)** Individual bubble plots for five phylotypes [*P. aeruginosa* **(B)**, *P. alcaliphila/P. oleovorans* **(C)**, *P. anguilliseptica* cluster **(D)**, *P. arsenicoxydans/P. mandelii* **(E)**, and *P. stutzeri* **(F)**], on the same nMDS, with absence of dot representing no detection of the phylotype and circle sizes proportional to untransformed relative abundance. *P. anguilliseptica* cluster includes species *P. anguilliseptica, P. peli*, and *P. guineae*. The scale is common to all plots.

### Sensitivity and Read Abundance Correlation Using NGS

Three *Pseudomonas*-negative samples (nuclease-free water, drinking water, and cooling tower water) were spiked with reference DNA of *P. aeruginosa* (10^1^ to 10^5^ genome copies) to determine the assay’s sensitivity and precision for quantitative measurements. For both nuclease-free water and drinking water samples, the detection limit was 10 genome copies per assay, while for the analyzed cooling tower water sample the detection limit was an order of magnitude higher (100 genome copies assay^-1^) (**Figure 2**). In addition, a strong correlation between Illumina MiSeq read abundance and the spiked in *P. aeruginosa* concentrations was observed for all samples and explained by a logarithmic regression model with the following coefficient determination (*R*^2^) values 0.96, 0.94, and 0.92 for the spiked nuclease-free water, drinking water, and cooling tower water samples, respectively.

### *Pseudomonas* Community Composition and Dynamics in Cooling Tower Water

The *Pseudomonas* community structure in a set of cooling tower water samples, sampled from January 2013 to December 2014, was investigated based on the genus-specific 16S rRNA gene V3-V4 region sequence discrimination. Illumina MiSeq sequencing produced a total of 1,177,496 merged paired-end reads for the whole dataset, of which 1,170,046 were classified to the genus *Pseudomonas* (99.4%). With the objective of removing potential spurious sequences, only OTUs with a number of reads higher than four were considered, reducing the total number of reads by 1.6% to 1,158,571, with a median of 41,261 reads. To assess if the sequencing depth per sample was adequate, rarefaction curves were plotted (**Supplementary Figure 5**). All samples curves showed a plateau, indicating that almost all of the *Pseudomonas* community diversity was captured. This was corroborated by a mean Good’s estimator of coverage of 97.8 ± 1.0%.

In our dataset, 43 *Pseudomonas* phylotypes were identified, of which 21 represented individual species that could be unambiguously classified, including the potentially pathogenic species *P. aeruginosa, P. otitidis*, and *P. stutzeri*. The *Pseudomonas* community in the cooling tower water samples was dominated by five phylotypes, representing 64.7% of the overall community (**Figure 3**). The phylotypes most commonly found were *P. aeruginosa* (18.5%), *P. arsenicoxydans/P. mandelii* (16.9%), *P. orientalis cluster* (12.0%), *P. alcaliphila/P. oleovorans* (8.8%), and *P. anguilliseptica* cluster (8.5%).

When performing phylogenetic grouping of the phylotypes (**Supplementary Figure 6**), *P. fluorescens* group was the most abundant (45.2%), mostly due to the presence of *P. arsenicoxydans/P. mandelii, P. orientalis* cluster, and *P. migulae/P. umsongensis*. The second most abundant was *P. aeruginosa* group (20.7%) with almost solely contribution of species *P. aeruginosa*. Other community relevant groups were *P. oleovorans* (9.3%) and *P. anguilliseptica* (9.0%). All remaining phylogenetic species groups represented less than 5% and were, contrarily to the others, at no time the most dominant.

The abundance of the major five phylotypes varied considerably among samples, ranging from 0 to more than 50% in a water sample. In addition, only 8 other phylotypes had a relative abundance higher than 1% within the whole community. Of the identified potentially pathogenic *Pseudomonas* species, *P. stutzeri* was the second most abundant after *P. aeruginosa* with an overall mean of 2.4% (maximum: 29.6%), followed by the less abundant *P. putida* and *P. otitidis*, with 0.2% (3.2%) and 0.1% (0.4%), correspondingly. When looking at the frequency of the phylotypes in the dataset, 17 of the 43 phylotypes were detected across a minimum of 75% (threshold used to define the *Pseudomonas* core community) of all cooling tower water samples. The 17 phylotypes, one being *P. aeruginosa*, represented 90.7% of the overall community, with a minimum 75.2% and a maximum of 99.9% of the total *Pseudomonas* community.

After classification of reads to *Pseudomonas* phylotypes, richness metrics revealed marked variation throughout the 2 years studied, with the number of phylotypes present in the cooling tower water ranging from 9 to 36 with a mean of 24 ± 8 phylotypes per sample (**Supplementary Table 5**). The observed *Pseudomonas* community richness was substantially reduced from September 2013 to January 2014 but gradually recovered over the following months. Shannon diversity index (*H’*) was estimated and showed considerable change in the cooling tower water system, with the two highest diversity values, i.e., June 2013 (4.16) and September 2014 (4.08), followed by reduced values in the succeeding month. These diversity reductions were due to a decrease in community evenness associated with a substantial increase in *P. aeruginosa* relative abundance.

In order to evaluate the *Pseudomonas* community structure and temporal variations, ordination analysis was performed (**Figure 4A**). By this analysis, no significant temporal trajectory with similar changes was observed (ANOSIM, *P* = 0.93). Instead, several abrupt changes in the *Pseudomonas* communities were detected during the studied time period and no significant differences in *Pseudomonas* communities between years occurred (ANOSIM, *P* = 0.27). Nevertheless, community gradually changed through time, within each year (ANOSIM, *R* = 0.44, *P* < 0.01). This shift was due to a change from a community mostly dominated by phylotypes *P. alcaliphila/P. oleovorans* (*P. oleovorans* group), *P. arsenicoxydans/P. mandelii* (*P. fluorescens* group), and *P. orientalis* (*P. fluorescens group*) to a community where *P. aeruginosa* (*P. aeruginosa* group) was the most abundant species, especially in 2013. Additionally, sample ordination was mostly explained by phylotypes *P. aeruginosa, P. alcaliphila/P. oleovorans, P. anguilliseptica* cluster, and *P. arsenicoxydans/P. mandelii* indicating the importance of these phylotypes in the community (**Figures 4B–E**). The temporal profiling of *Pseudomonas* communities, after species phylogenetic grouping, revealed a very significant resemblance to the previous analysis (RELATE, *r_s_* = 0.82, *P* < 0.05), pointing toward little contribution of temporal shifts within each phylogenetic group to elucidate community dynamics.

The temporal shifts in the community structure over the studied time period showed significant correlations with the abiotic factors conductivity, temperature, and water exchange rate (distLM, *P* < 0.05). The increased value of the parameters previously mentioned were temporally associated with dominance of *P. aeruginosa*, particularly in 2013. Yet, the 2-year community dynamics observed could not be explained by the environmental and operational parameters measured (RELATE, *P* = 0.2).

**FIGURE 5:**
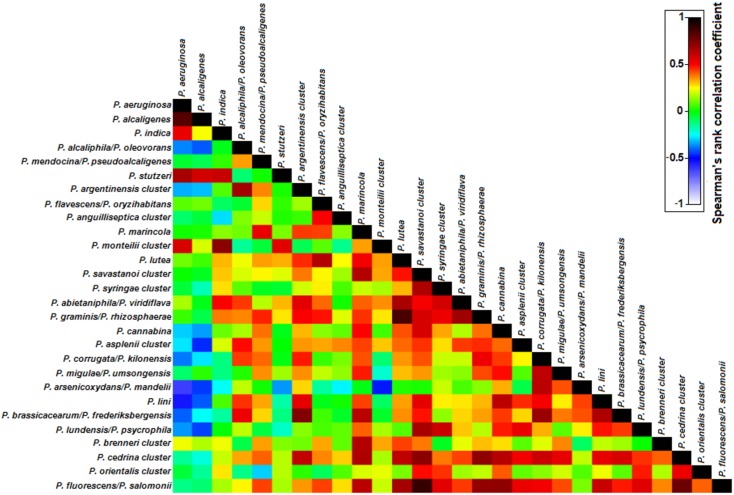
Spearman’s rank correlation matrix of the *Pseudomonas* phylotypes in the analyzed cooling tower water samples. Phylotypes listed were detected in at least 50% of the water samples. The colors of the bar indicate the nature and degree of correlation with 1 indicating maximum positive association and -1 indicating maximum negative correlation between two phylotypes.

Despite being simultaneously detected in most water samples a negative correlation in their abundances was observed between *P. aeruginosa* and both *P. alcaliphila/P. oleovorans* (*r_s_* = -0.37, *P* < 0.05) and *P. arsenicoxydans/P. mandelii*, (*r_s_* = -0.62, *P* < 0.01), suggesting that their high abundance might prevent dominance of *P. aeruginosa* in the community (**Figure 5**). Next, we focused on phylotypes showing positive correlation patterns in relative abundance with *P. aeruginosa* (**Figure 5**). These included other species of *P. aeruginosa* phylogenetic group, i.e., *P. alcaligenes* (*r_s_* = 0.83, *P* < 0.01) and *P. indica* (*r_s_* = 0.53, *P* < 0.01); as well as *P. monteilii* cluster (*P. putida* group; *r_s_* = 0.57, *P* < 0.01) and potentially pathogenic *P. stutzeri* (*P. stutzeri* group; *r_s_* = 0.67, *P* < 0.01), supporting the nMDS observations (**Figure 4F**). These phylotypes were characterized by a substantial reduction in their abundance after November 2013 with a progressive recovery of their community position from August 2014 on.

By comparing the dynamics of *P. aeruginosa* with the ones of other potentially pathogenic bacteria genera (pan-bacterial approach) and *Legionella* species (genus-specific approach) that were previously studied in the same cooling tower system (Pereira et al., 2017b), we observed a significant temporal association with taxa *Acinetobacter, Bacillus, Enterobacter/Klebsiella, Escherichia/Shigella*, and *Staphylococcus* (SIMPROF III, *P* < 0.05); and clinically *Legionella* relevant species *L. pneumophila* and *L. feelei* (SIMPROF III, *P* < 0.05).

### Absolute Quantification of *Pseudomonas* Species in Cooling Tower Water

**FIGURE 6:**
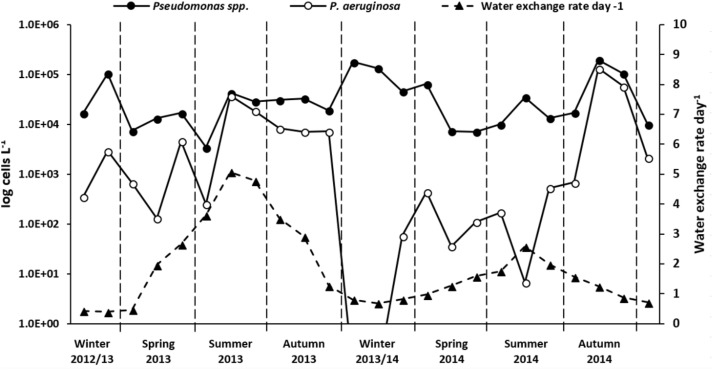
Absolute *Pseudomonas* species and *P. aeruginosa* quantification results from January 2013 to December 2014. Data are given as cells per liter of cooling tower water. Note the log transformation of left y-axis. *Pseudomonas* species cell counts (filled circle, continuous line) represent mean after replicates (*n* = 3) analysis by qPCR. *P. aeruginosa* cell counts (empty circle, solid line) calculated as a function of relative abundances obtained from NGS *Pseudomonas* community analysis. Water exchange rate values per day (filled triangles, dashed line) are also shown. Winter, December to February; spring, March to May; summer, June to August; autumn, September to November.

*Pseudomonas* species absolute abundance in cooling tower water samples was quantified by a qPCR assay using the same primer pair as used for NGS. When applying this assay, *Pseudomonas* species, as expected, were detected in every analyzed sample, with an overall median value of 2.4 × 10^4^ cells L^-1^. The quantitative data showed a temporal variation with monthly fluctuation (**Figure 6** and **Supplementary Table 6**). This variation was independent of the environmental and operational parameters assessed. No significant annual variation was observed (*t*-test, *P* = 0.81), with *Pseudomonas* species median concentration values of 2.4 × 10^4^ cells L^-1^ in 2013 and 2.6 × 10^3^ cells L^-1^ in 2014. Yet, steep monthly *Pseudomonas* species abundance increments were mostly detected during summer and winter. The *Pseudomonas* species abundance was higher in winter (9.0 × 10^4^ cells L^-1^) than in autumn (median = 6.7 × 10^4^ cells L^-1^), summer (2.2 × 10^4^ cells L^-1^) or spring (1.9 × 10^4^ cells L^-1^).

In order to apply NGS for absolute *Pseudomonas* species concentration determination and to do a cross-platform comparison between the optimized and validated *Pseudomonas*-specific NGS and qPCR assays, 12 cooling tower water samples, monthly collected in 2014, were analyzed. When comparing both methods, regarding their quantitative absolute measurement of *Pseudomonas* species concentration in the studied samples, a strong correlation (Pearson’s *r* = 0.85, *p*<0.05) was shown (**Supplementary Figure 7**).

Since NGS read counts allowed a precise quantification of *P. aeruginosa* along a dilution range (**Figure 2**) and showed a strong correlation with the absolute numbers of a validated qPCR assay (**Supplementary Figure 7**), *P. aeruginosa* absolute abundance in cooling tower samples were also calculated, as a function of the relative abundances obtained with NGS (**Figure 6** and **Supplementary Table 6**). *P. aeruginosa* concentration varied markedly in the system, from not being detected (December 2013 and January 2014) to values as high as 1.3 × 10^5^ cells L^-1^ (October 2014). *P. aeruginosa* absolute numbers were greater in 2013 (median = 3.8 × 10^3^ cells L^-1^) than in 2014 (3.0 × 10^2^ cells L^-1^; *t*-test, *P* = 0.18); with higher concentrations in summer/autumn 2013 and autumn 2014. The monthly variation of *P. aeruginosa* cell numbers showed a significant positive correlation with conductivity (*r_s_* = 0.64, *P* < 0.05) and total hardness (*r_s_* = 0.41, *P* < 0.05) along the studied timeline; and with water exchange rate (*r_s_* = 0.67, *P* < 0.05) and temperature (*r_s_* = 0.61, *P* < 0.05) solely in 2013.

## Discussion

### Validation of *Pseudomonas*-Specific NGS Assay

Freshwater is the natural reservoir for *Pseudomonas* species and the main source of human exposure. Therefore, assessment of *Pseudomonas* species composition and dynamics, and the detection of potentially pathogenic species, such as *P. aeruginosa*, is of scientific interest and public health importance (Anaissie et al., 2002; Trautmann et al., 2005; Mena and Gerba, 2009). With this in mind, we developed an Illumina-based NGS study and directly applied it, in combination with a validated qPCR assay, to a variety of freshwater samples to investigate the abundance and presence of *Pseudomonas* species. We opted to design and apply a more targeted genus-specific approach as previous studies have shown that group-specific NGS studies allow, when compared to pan-bacterial approaches, not only a better characterization of specific taxonomic or functional groups but also a higher detection and more precise quantification of low-abundant bacteria (Walker et al., 2015; Pereira et al., 2017a). To the best of our knowledge, this is the first 16S rRNA gene-based Illumina NGS assay developed for the detailed study of the *Pseudomonas* community.

Within the genus *Pseudomonas*, 16S rRNA gene-based assays have the major limitation of a low resolution at the interspecies level, however, several described species, including *P. aeruginosa*, can still be clearly distinguished (Moore et al., 1996; Anzai et al., 2000; Bodilis et al., 2012). In addition, interpretation of data at the phylogenetic species group level is of clinical and environmental significance (Matthijs et al., 2013; Remold et al., 2015). In order to overcome the low discriminatory power of the 16S rRNA gene, other genes encoding highly conserved housekeeping proteins, such as the σ^70^ subunit of RNA polymerase (*rpoD*), have been recommended previously (Mulet et al., 2010). Yet, a recent study from Sánchez et al. (2014), applying a novel *rpoD*-based NGS approach to one river water sample for assessment of *Pseudomonas* diversity, showed considerably low target specificity, reflecting the limitations of using a gene with no robust and curated accessible sequence database. Hence, in our opinion, approaches based on other marker genes, presently, cannot be accurately and successfully pursued with ease using platforms with a limiting maximum fragment length.

For a thorough validation of an NGS assay for environmental and clinical purposes, assessment of its specificity, repeatability, accuracy and quantitative precision is of critical importance. The designed molecular approach revealed a superior coverage of the targeted taxa with less sequencing depth due to a high *in situ* specificity (> 98%) in the studied freshwater samples. These observations show consistency between *in silico* and *in situ* data and are in agreement with observations from soil samples with the same primer set (Bergmark et al., 2012). The potential amplification of closely related taxa *Azomonas* and *Azotobacter* makes the specificity of the assay dependent on the microbial composition of the samples analyzed. However, considering the regularly low abundance of these genera in the environment and the taxonomy information provided by the NGS approach, a detrimental effect on the robustness of the assay is unlikely with adequate sequencing depth.

When analyzing the accuracy and quality of the *P. aeruginosa* sequences generated by our NGS approach, results showed a low global error rate (0.3%), with most of the reads being error-free. The error frequency and sequence quality observed are within the range of what has been described by other studies using distinct Illumina-based approaches (0.2–0.5%) (Schirmer et al., 2015; Pereira et al., 2017a). Substitutions were the most frequent and the most ubiquitously distributed errors, supporting identical findings in previous studies with Illumina platforms (Loman et al., 2012; Schirmer et al., 2015; Pereira et al., 2017a). Moreover, the most commonly detected substitutions were transitions (A to G, G to A, C to T, T to C), which is in agreement with known results (D’Amore et al., 2016; Pereira et al., 2017a). In addition, the substantial error rate per base range (0.07–0.7%) highlights the existence of error hotspots which are almost definitely sequence and motif-dependent errors (Nakamura et al., 2011; Schirmer et al., 2015). As a result of the high accuracy and quality of the approach, the probability of species or phylotype misidentification is low, allowing our assay to be confidently applied to environmental research and diagnostics.

When looking at the technical variation introduced at the library preparation and sequencing steps, discrepancies in the *Pseudomonas* structure, independent of sequencing effort, existed among replicates but were not significant (ANOSIM, *P* > 0.05). In general, samples with greater *Pseudomonas* community diversity and lower *Pseudomonas* template concentration revealed higher inter-replicate variability, being our results consistent with previous findings (Polz and Cavanaugh, 1998; Kennedy et al., 2014). The dissimilarities observed among replicates in both abundant and rare taxa reveal that replication with appropriate sequencing depth might be desirable to reduce misrepresentative outliers, minimize within-sample heterogeneity and allow for a more reliable detection of low-abundant taxa.

In addition, the developed approach showed a sample-independent precise quantification of *P. aeruginosa* using a standard series of defined genomic standards. This is consistent with other observations using a similar approach for detection and quantification of *L. pneumophila* (Pereira et al., 2017a). Nonetheless, sensitivity of the assay in cooling tower water was lower than in drinking water and nuclease-free water, which confirms a higher probability of an inhibitory effect being present in these water samples, leading to underestimation or non-detection of the target (Miyamoto et al., 1997).

### *Pseudomonas* Community Profiling in Cooling Tower Water

The validated and optimized Illumina-based NGS assay targeting *Pseudomonas* species was applied to water from a cooling tower, known to provide a unique environment for bacterial growth, biofilm formation and dissemination (Liu et al., 2009). For all we know, *Pseudomona*s communities have not been studied in detail in cooling tower environments, though *Pseudomonas* are important biofilm producers and corrosion promoters, and their infections have been related with aerosol transmission (Döring et al., 1991; Vallés et al., 2004; Blasco et al., 2008; Liu et al., 2009). Our study identified 43 phylotypes in spite of the lack of resolution provided by the fragment amplified and the consequent underestimation of the true diversity and richness of the community. Moreover, an important temporal core community, representing 75% or more of the total *Pseudomonas* community in each sample, primarily consisting of the phylotypes *P. aeruginosa, P. arsenicoxydans*/*P. mandelii, P. orientalis* cluster, *P. alcaliphila*/*P. oleovorans*, and *P. anguilliseptica* cluster was observed. That is indicative of a very stable community persisting in the freshwater environment, where mostly relative abundances, rather than taxa, shift in response to heterogeneity of biotic and abiotic factors along time.

With respect to the *Pseudomonas* community composition and in agreement to findings in river water (Matthijs et al., 2013; Sánchez et al., 2014), most of the classified reads belonged to species and species clusters within the *P. fluorescens* group (45.2%). Several studies have reported a high abundance of *P. putida* species group in environmental samples (Matthijs et al., 2013; Remold et al., 2011, 2015) but this group had little relevance in the studied cooling tower (3.3%). *P. aeruginosa* was the most abundant phylotype in the community (18.5%) and by far the most predominant pathogenic *Pseudomonas* species, strengthening the focus of detection of this species in freshwater microbial quality monitoring.

Ordination analysis revealed that the dissimilarity between the *Pseudomonas* communities was strongly influenced by the phylotypes *P. aeruginosa, P. arsenicoxydans/P. mandelii*, and *P. alcaliphila/P. oleovorans*, revealing not only the importance of these phylotypes in the community but also a clear dominant species shift within each year. Transposing the results to the cooling tower system from the studies in river systems that detected a predominance of *P. aeruginosa* in polluted areas (Pirnay et al., 2005; Matthijs et al., 2013), we can hypothesize that there is a decrease of the microbial water quality in the system throughout the year and especially in 2013. This could be explained by an accumulation of sediments and bacterial slims and biofilms, in a process known as fouling (Jenner et al., 1998). The suggested microbial water quality link is strengthened by the temporal association of *P. aeruginosa* with potentially pathogenic and biofilm-associated bacteria, i.e., *Acinetobacter, Staphylococcus, Enterobacter/Klebsiella, Escherichia/Shigella*, and *L. pneumophila.* In addition, the turnover to a *P. aeruginosa*-dominant community was linked to increased values of conductivity, which frequently is an important indicator of water quality. Nonetheless, the study revealed that the environmental and operational parameters assessed did not explain the *Pseudomonas* community temporal dynamics in the cooling system, emphasizing the complexity of these man-made systems and the multitude of potential biotic and abiotic drivers in these ecosystems.

The study of negative associations and co-occurrence of pathogenic bacteria with other bacteria, as well as the understanding of the clashing/common metabolic pathways and the distinctive/cooperative traits that undermine/determine virulence are of great importance (Griffin et al., 2004). *P. aeruginosa* showed a negative association with dominant phylotypes *P. arsenicoxydans/P. mandelii* (*P. fluorescens* group) and *P. alcaliphila/P. oleovorans* (*P. oleovorans* group) suggesting that interspecific competition, without exclusion, for a similar niche exists and high abundance of these phylotypes may out-compete *P. aeruginosa* and have a detrimental effect on the species growth and dominance in the cooling tower water. Moreover, *P. aeruginosa* co-occurred with phylotypes of *P. aeruginosa, P. putida*, and *P. stutzeri* groups; but showed a negative association with several phylotypes of *P. fluorescens* group. These results not only suggest that a phylogenetic proximity might reflect collaborative associations and complementary functional traits (Griffin et al., 2004), but also show co-existence of opportunistic bacterial pathogens that need to be further investigated. Furthermore, a very substantial increment of *P. aeruginosa* abundance was observed when a highly diverse *Pseudomonas* community was previously present. This finding suggests that in high diverse samples with absence of a dominant phylotype, *P. aeruginosa* could take the opportunity to become prevalent. *P. aeruginosa* due to its high metabolic and nutritional versatility might have a competitive edge, an increased ability to rapidly colonize a niche that is potentially available, as a result of diversification. This knowledge may provide us with an opportunity to better track and control *P. aeruginosa* in freshwater environments, which should be, assessed in more detail in future studies. These studies should not only address the *Pseudomonas* communities but as well the impact of the whole bacterial community and other trophic levels, such as grazers and viruses, on the abundance and presence of *P. aeruginosa* (Weinbauer and Höfle, 1998).

### Absolute Quantification of *Pseudomonas* Species in Cooling Tower Water

Next generation sequencing read counts are generally not taken in consideration for absolute measurement of a taxon, undermining the evaluation of NGS robustness for absolute quantification. Here, we found a good correlation between *Pseudomonas* species absolute quantification using NGS read numbers and qPCR quantitation cycle values. These results strongly suggest that our novel assay can be used as an alternative to qPCR, for simultaneous determination of *Pseudomonas* species abundances and absolute quantification of *P. aeruginosa*. Yet, as qPCR is the gold standard for quantitative analysis, we used the same set of primers and assessed the absolute quantification of *Pseudomonas* in the cooling tower system for all monthly samples during the years 2013 and 2014.

Though the genus *Pseudomonas* comprises several pathogenic species and has previously been proposed as an indicator of potential bacterial regrowth in freshwater systems (Ribas et al., 2000), qPCR-based studies on the distribution of *Pseudomonas* in freshwater samples have been missing, and little is known regarding temporal and spatial variation. The absolute abundance of *Pseudomonas* in our cooling tower system (most values ranged from 10^4^ to 10^5^ cells L^-1^) was significantly lower when compared to a report of 10^4^ cfu ml^-1^ in a cooling tower system in Valencia (Spain) (Blasco et al., 2008). The density values detected were more in the range of what has been reported in drinking water (Ribas et al., 2000; Vaz-Moreira et al., 2012) and freshwater reservoirs (Flores Ribeiro et al., 2014). This seems to be indicative that the cooling tower studied is not a very favorable environment for proliferative growth of *Pseudomonas*. However, further studies on different cooling tower systems, and on the characteristics and importance of make-up water would be required to confirm this. For instance, the fact that the concentration of dissolved organic carbon (DOC) in the drinking water used as make up water is low (2 mg C L^-1^) could be a contributing factor. The above-mentioned study in Valencia (Spain) also revealed significantly lower counts in winter (Blasco et al., 2008), opposing our findings. Overall, this reveals very distinct behaviors and absolute concentration ranges among different cooling tower systems that need to be better understood.

The absolute abundance of *P. aeruginosa* in the cooling tower system, inferred from NGS relative abundances, varied substantially, with most values lower than 10^3^ cells L^-1^, but reaching values up to 10^5^ cells L^-1^ (autumn 2014). The concentration of *P. aeruginosa* in most periods is comparable to the ones described in drinking water distribution systems (Wang et al., 2012; Lu et al., 2016) and sediments (Lu et al., 2015; Qin et al., 2017). These results stress the need to better comprehend how distinct temperature-driven water exchange rates and make-up water sources can influence the absolute abundance of *P. aeruginosa* in a man-made freshwater system.

This study has revealed the importance of NGS as a tool to carve the way toward a better understanding of *Pseudomonas* community composition and dynamics in freshwater environments and highlights the potential of this assay for application to freshwater quality monitoring. However, little is known for most *Pseudomonas* species and further investigations regarding their genetics, ecology, metabolic networks and niche partitioning are required.

## Author Contributions

RP designed the study, performed the experiments, and analyzed the data. JP processed the raw NGS data. DM provided the river water samples. IB provided the real-time analysis and environmental and operational data. MH designed the study. RP and MH wrote the manuscript. All authors read, reviewed, and approved the manuscript.

## Conflict of Interest Statement

JP was employed by bioinformatics company Ribocon GmbH (Bremen, Germany). The remaining authors declare that the research was conducted in the absence of any commercial or financial relationships that could be construed as a potential conflict of interest.

## Footnotes

1 https://rrndb.umms.med.umich.edu
