# Supplementary material for: Pseudomonas-Specific NGS Assay Provides Insight Into Abundance and Dynamics of Pseudomonas Species Including P. aeruginosa in a Cooling Tower
Source: Front Microbiol. 2018 Aug 21;9:1958. doi: 10.3389/fmicb.2018.01958 (PMC6110898; doi:10.3389/fmicb.2018.01958)
Supplement: Supplementary file 1 [file Data_Sheet_1.DOCX]

***supplementary Material***

***Pseudomonas*-specific NGS assay provides insight into abundance and dynamics of *Pseudomonas* species including *P. aeruginosa* in a cooling tower**

**Rui P.A. Pereira, Jörg Peplies, Douglas Mushi, Ingrid Brettar, Manfred G. Höfle***

***Correspondence:**

Manfred G. Höfle

manfred.hoefle@helmholtz-hzi.de

1. **Supplementary Figures and Tables**

**1.1 Supplementary Figures**

**
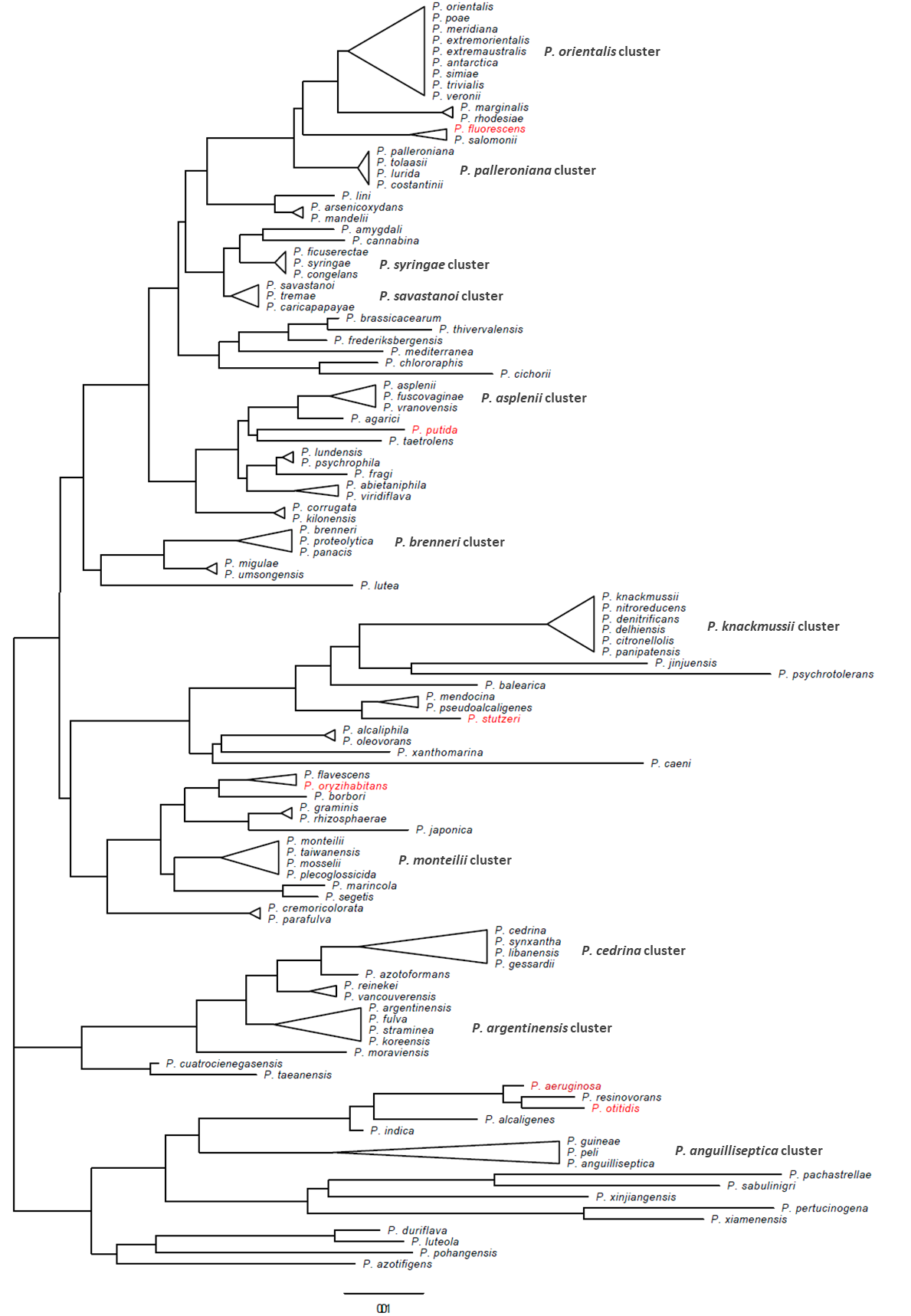
**

**Supplementary Figure 1. Phylogenetic resolution of main *Pseudomonas* species based on the 16S rRNA gene V3-V4 region amplified by primer pair 434F/665R.** Phylogenetic tree as a graphic representation of the distance matrix calculated using the Kimura 2-parameter method and generated by Neighbor-Joining. *Pseudomonas* species known to have been associated with human disease are highlighted in red. Please note that phylogeny should not be inferred with this tree.


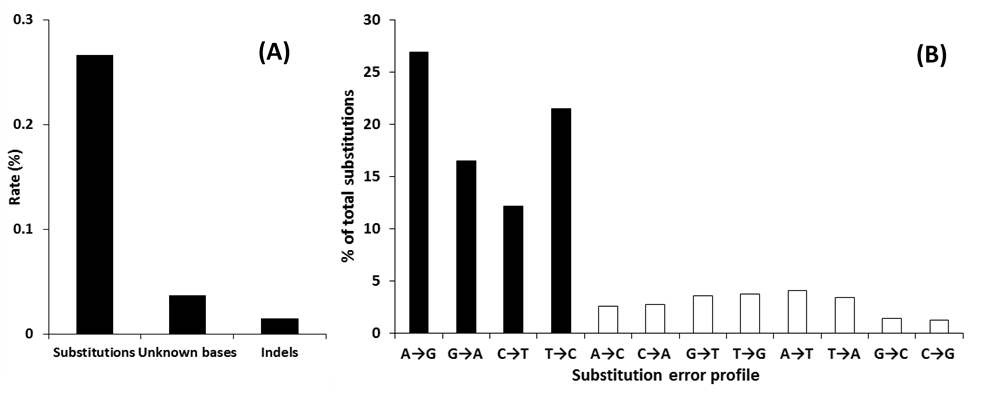


**Supplementary Figure 2. Error rate profiling.** Analysis of the NGS library preparation and Illumina MiSeq amplification and sequencing using 15,000 reads of *P. aeruginosa* DSM 50071^T^. Bars represent the mean value percentage of the different types of errors per position (**A**) and the substitution percentage breakdown to the different nucleotide substitutions (nucleotide expected → nucleotide detected) (**B**). Black-colored and white-colored bars represent transition and transversion substitutions, in that order. Substitution profile preferences differed significantly with substitutions A to G (26.9%), T to C (21.6%), G to A (16.5%) and C to T (12.1%) the most frequent. Each of the remaining substitutions represented less than 5% of the total substitutions.


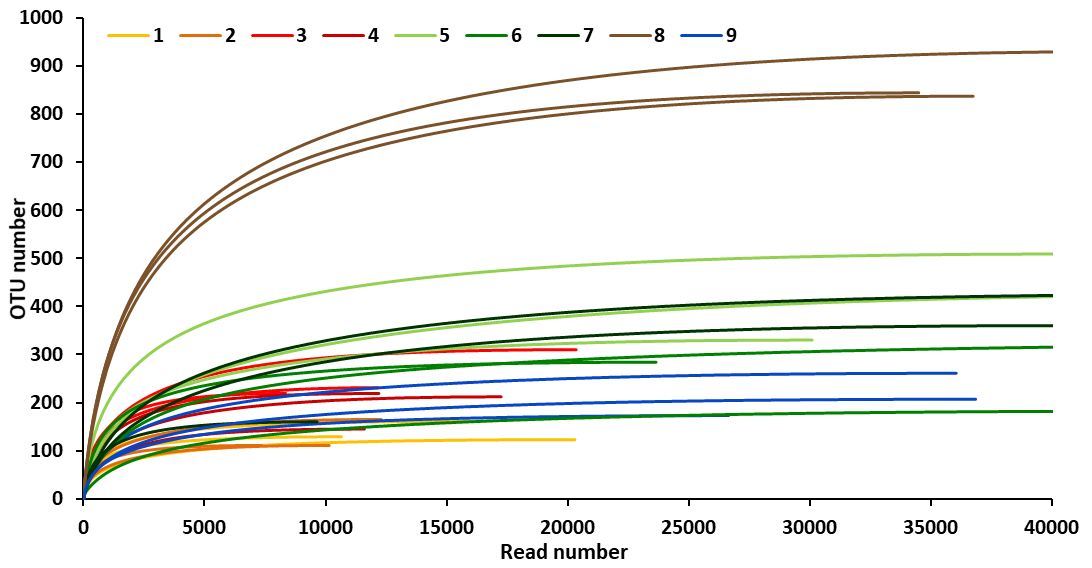


**Supplementary Figure 3. Rarefaction curves for 27 *Pseudomonas* communities of nine different water samples (four cooling tower water, three river water and one drinking water).**


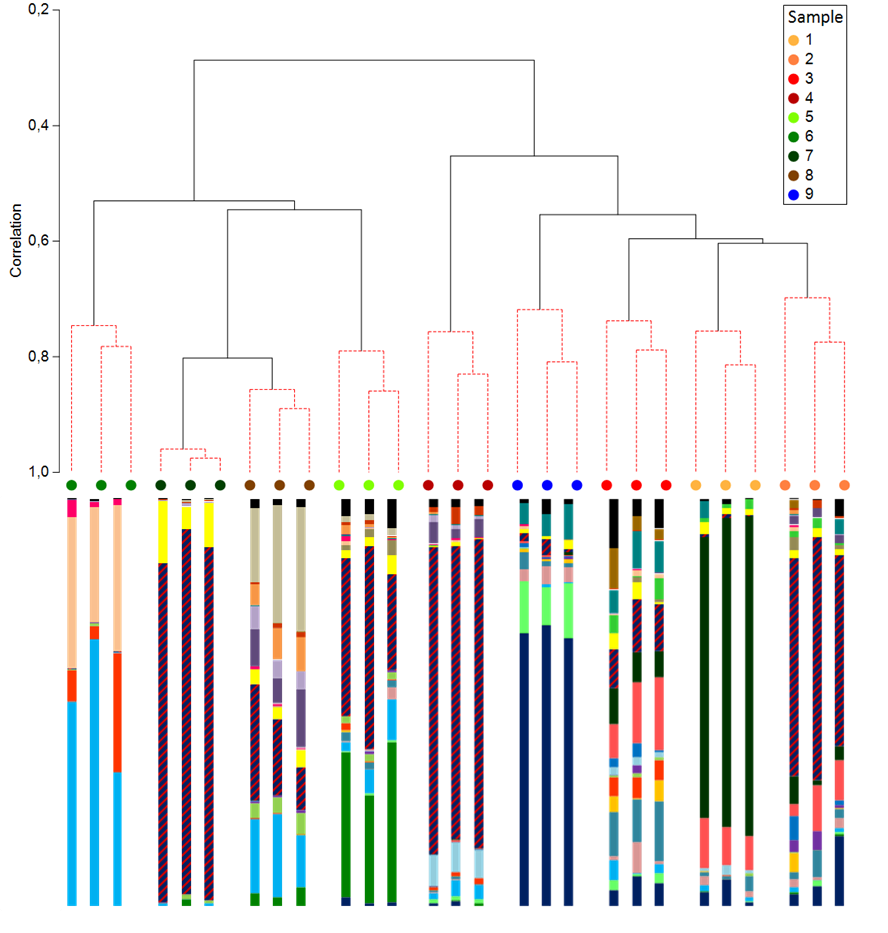


**Supplementary Figure 4. Dendrograms showing group-average hierarchical clustering of triplicates of nine freshwater samples using weighted Spearman rank correlation.** SIMPROF test was performed with 999 permutations. Red dashed lines represent groups that do not significantly differ. The bar graph graphically represents the relative abundance of *Pseudomonas* phylotypes for each sample. *P. aeruginosa* abundances are represented by a hatched pattern. Cooling tower water (samples 1 to 4); river water (5 to 8) and drinking water (9).


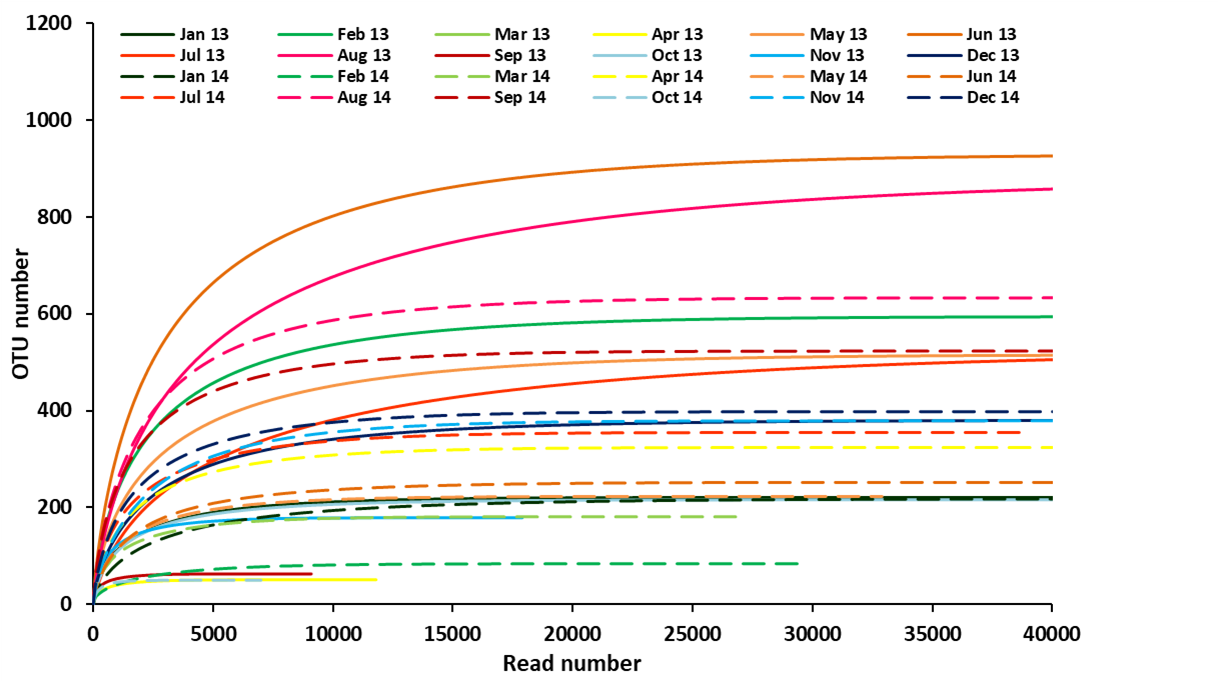


**Supplementary Figure 5.** **Rarefaction curves for *Pseudomonas* communities of cooling tower water samples, monthly collected from January 2013 to December 2014.**

**
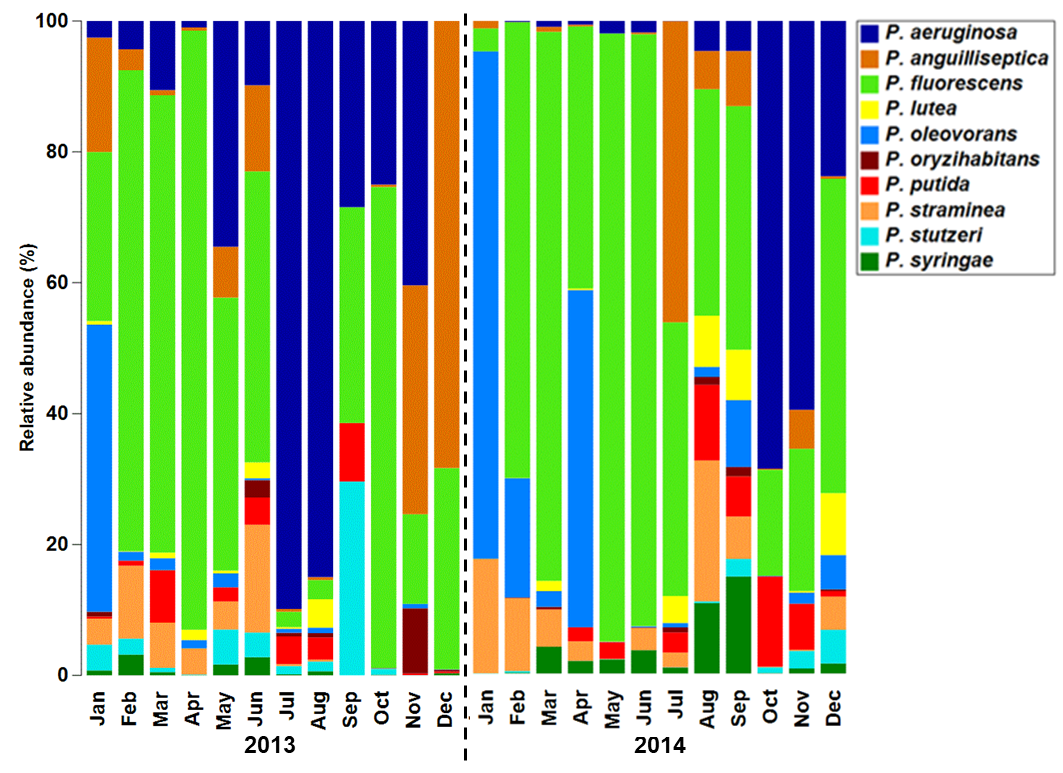
**

**Supplementary Figure 6. *Pseudomonas* community composition of cooling tower water samples assessed by 16S rRNA gene amplicon sequencing with Illumina MiSeq.** Bar chart showing relative abundance of *Pseudomonas* phylogenetic groups (%). P. fluorescens species group was the most abundant (45.2%), followed by *P. aeruginosa* (20.7%), *P. oleovorans* (9.3%), *P. anguilliseptica* (9.0%), *P. straminea* (5.2%), *P. putida* (3.3%), *P. stutzeri* (2.6%), *P. syringae* (2.1%), *P. lutea* (1.7%) and *P. oryzihabitans* (0.8%). Samples collected monthly in 2013 and 2014 are separated by year with a dashed line. Jan, January; Feb, February; Mar, March; Apr, April; Jun, June; Jul, July; Oct, October; Nov, November; Dec, December.


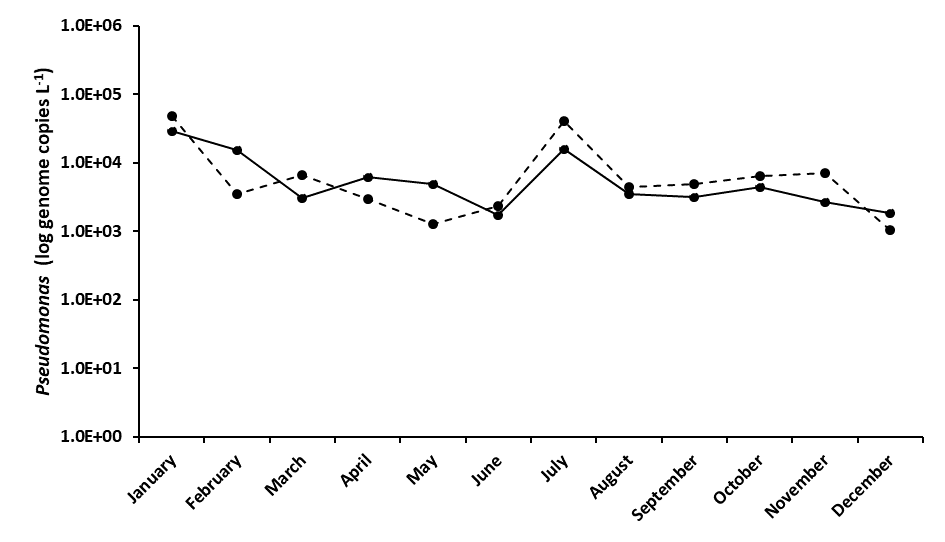


**Supplementary Figure 7. Measurement of *Pseudomonas* species concentrations with NGS (solid line) and qPCR (dashed line).** Data are given as *Pseudomonas* genome copies per liter of cooling tower water. 12 cooling tower water samples monthly sampled in 2014 were analyzed. Values represent mean after replicates (n = 3) analysis. Note the log transformation of y-axis.

**1.2 Supplementary Tables**

**Supplementary Table 1A. Physicochemical and biological parameters for cooling tower water from January to December 2013^a^.** n.d., not detected; n.a. not assessed.

| **Parameters** | **Jan** | **Feb** | **Mar** | **Apr** | **May** | **Jun** | **Jul** | **Aug** | **Sep** | **Oct** | **Nov** | **Dec** |
| --- | --- | --- | --- | --- | --- | --- | --- | --- | --- | --- | --- | --- |
| **Temperature (°C)** | 11.0 | 8.8 | 9.3 | 9.5 | 24.9 | 24.2 | 25.5 | 27.5 | 27.5 | 24.8 | 10.9 | 11.9 |
| **pH** | 8.59 | 8.59 | 8.60 | 8.61 | 8.62 | 8.93 | 9.04 | 8.89 | 8.89 | 8.28 | 8.32 | 8.19 |
| **Conductivity (µS cm^-1^)** | 419 | 440 | 439 | 349 | 394 | 413 | 417 | 441 | 441 | 430 | 401 | 386 |
| **Ammonia (mg L^-1^)** | 0.02 | 0.04 | 0.07 | 0.40 | 0.02 | 0.03 | <0.01 | 0.03 | <0.01 | 0.03 | 0.06 | n.a. |
| **Chlorine (mg L^-1^)** | 0.1 | 0.1 | 0.5 | 0.1 | n.d. | 0.1 | 0.1 | 0.1 | 0.1 | 0.1 | 1.0 | 0.5 |
| **Iron (mg L^-1^)** | 1.32 | 1.65 | 1.65 | 1.32 | 0.66 | 0.33 | 0.33 | n.d. | n.d. | 0.66 | 0.99 | 1.65 |
| **Nitrate (mg L^-1^)** | 6.00 | n.d. | 19.00 | 14.00 | 12.00 | 10.00 | 13.00 | 17.00 | 16.00 | 14.00 | 10.00 | n.a. |
| **Phosphate (mg L^-1^)** | 1.30 | 1.50 | 1.60 | 1.90 | 0.90 | 0.80 | 0.70 | 0.60 | 0.60 | 1.00 | 1.20 | 1.50 |
| **Zinc (mg L^-1^)** | 0.48 | 0.40 | 0.40 | 0.40 | 0.24 | 0.24 | 0.24 | 0.08 | 0.40 | 0.24 | 0.40 | 0.32 |
| **Total hardness (°dH)** | 9.31 | 9.59 | 9.59 | 7.26 | 8.80 | 7.07 | 8.93 | 9.26 | 8.42 | 9.12 | 9.54 | 8.15 |
| **TCC (cells L^-1^)** | 1.8x10^9^ | 1.2x10^9^ | 5.1x10^8^ | 9.3x10^8^ | 2.0x10^9^ | 6.1x10^8^ | 9.9x10^8^ | 4.0x10^8^ | 4.8x10^8^ | 1.2x10^9^ | 7.5x10^8^ | 9.7x10^8^ |

**Supplementary Table 1B. Physicochemical and biological parameters for cooling tower water from January to December 2014^a^.** n.d., not detected; n.a., not assessed.

| **Parameters** | **Jan** | **Feb** | **Mar** | **Apr** | **May** | **Jun** | **Jul** | **Aug** | **Sep** | **Oct** | **Nov** | **Dec** |
| --- | --- | --- | --- | --- | --- | --- | --- | --- | --- | --- | --- | --- |
| **Temperature (°C)** | 21.6 | 8.7 | 20.5 | 22.0 | 21.2 | 22.9 | 22.4 | 22.0 | 21.4 | 22.2 | 21.6 | 9.3 |
| **pH** | 8.62 | 8.75 | 8.58 | 8.53 | 8.70 | 8.61 | 8.82 | 8.41 | 8.31 | 8.52 | 8.45 | 8.32 |
| **Conductivity (µS cm^-1^)** | 388 | 376 | 398 | 347 | 358 | 372 | 387 | 355 | 336 | 436 | 425 | 487 |
| **Ammonia (mg L^-1^)** | 0.08 | n.a. | 0.08 | 0.07 | 0.33 | 0.02 | 0.04 | 0.08 | 0.12 | n.a. | 0.07 | 0.13 |
| **Chlorine (mg L^-1^)** | 0.1 | 0.1 | 0.5 | 0.5 | 0.5 | 0.5 | 0.5 | 0.5 | 0.5 | 0.1 | 0.5 | 1.0 |
| **Iron (mg L^-1^)** | 1.32 | 1.65 | 1.32 | 0.99 | 0.33 | 1.32 | 1.32 | 0.33 | 2.97 | 1.98 | 0.99 | 1.98 |
| **Nitrate (mg L^-1^)** | 12.00 | n.a. | 14.00 | 13.00 | 12.00 | 12.00 | 13.00 | 11.00 | 10.00 | n.a. | 12.00 | 14.00 |
| **Phosphate (mg L^-1^)** | 0.90 | 1.30 | 1.20 | 0.90 | 0.80 | 1.00 | 0.70 | 0.70 | 3.30 | 3.10 | 2.10 | 2.40 |
| **Zinc (mg L^-1^)** | 1.12 | 0.96 | 1.76 | 1.12 | 0.48 | 0.88 | 1.44 | 0.64 | 0.80 | 0.88 | 0.72 | 0.72 |
| **Total hardness (°dH)** | 8.05 | 7.82 | 8.65 | 7.54 | 7.91 | 8.33 | 8.92 | 8.61 | 7.21 | 7.91 | 8.56 | 10.52 |
| **TCC (cells L^-1^)** | 6.9x10^8^ | 1.3x10^9^ | 9.0x10^8^ | 7.1x10^8^ | 4.5x10^8^ | 4.3x10^8^ | 6.1x10^8^ | 6.3x10^8^ | 1.2x10^9^ | 8.7x10^8^ | 1.5x10^9^ | 1.6x10^9^ |

^a^ Temperature was determined using the digital hand-multimeter 500 Ktype (Conrad Electronic SE, Hirschau, Germany) and pH was measured by a laboratory digital pH meter (Knick, Berlin, Germany). The Multiline P4 universal meter (WTW, Weilheim, Germany) was used to measure conductivity. Photometric analysis using Nanocolor tube tests (Macherey-Nagel, Düren, Germany) was performed to quantify ammonia (NH_4_^+^), calcium (Ca^2+^), iron (Fe^2+^/Fe^3+^), magnesium (Mg^2+^), nitrate (NO_3_^-^), phosphate (PO_4_^3-^) and zinc (Zn^2+^). Chlorine (Cl_2_) was determined by Quantofix test strips (Macherey-Nagel, Düren, Germany).

**Supplementary Table 2A. Cooling tower operational parameters rates in year 2013.**

| **Parameters** | **Jan** | **Feb** | **Mar** | **Apr** | **May** | **Jun** | **Jul** | **Aug** | **Sept** | **Oct** | **Nov** | **Dec** |
| --- | --- | --- | --- | --- | --- | --- | --- | --- | --- | --- | --- | --- |
| **Water exchange rate day^-1^** | 0.43 | 0.40 | 0.47 | 1.96 | 2.66 | 3.63 | 5.07 | 4.78 | 3.52 | 2.91 | 1.27 | 0.80 |
| **Make-up water (m^3^ day^-1^)** | 9.36 | 8.71 | 10.32 | 43.13 | 58.48 | 79.77 | 111.51 | 105.26 | 77.33 | 64.07 | 27.87 | 17.61 |
| **Draw off water (m^3^ day^-1^)** | 2.84 | 3.14 | 3.58 | 15.47 | 21.48 | 26.77 | 38.77 | 36.39 | 26.93 | 22.45 | 8.87 | 6.16 |
| **Evaporation (m^3^ day^-1^)** | 6.52 | 5.57 | 6.74 | 27.67 | 37.00 | 53.00 | 72.74 | 68.87 | 50.40 | 41.61 | 19.00 | 11.45 |

**Supplementary Table 2B. Cooling tower operational parameters rates in year 2014.**

| **Parameters** | **Jan** | **Feb** | **Mar** | **Apr** | **May** | **Jun** | **Jul** | **Aug** | **Sept** | **Oct** | **Nov** | **Dec** |
| --- | --- | --- | --- | --- | --- | --- | --- | --- | --- | --- | --- | --- |
| **Water exchange rate day^-1^** | 0.68 | 0.82 | 0.97 | 1.27 | 1.59 | 1.76 | 2.58 | 1.98 | 1.57 | 1.25 | 0.87 | 0.71 |
| **Make-up water (m^3^ day^-1^)** | 14.97 | 17.96 | 21.29 | 27.87 | 34.87 | 38.63 | 56.84 | 43.55 | 34.57 | 27.42 | 19.17 | 15.71 |
| **Draw off water (m^3^ day^-1^)** | 4.97 | 5.61 | 7.36 | 10.37 | 12.16 | 13.20 | 21.74 | 11.97 | 13.33 | 9.36 | 6.33 | 5.29 |
| **Evaporation (m^3^ day^-1^)** | 10.00 | 12.36 | 13.94 | 17.50 | 22.71 | 25.43 | 35.10 | 31.58 | 21.23 | 18.07 | 12.83 | 10.42 |

**Supplementary Table 3A. Nucleotide sequences of *Pseudomonas* genus-specific NGS primers, targeting the 16S rRNA gene, used in the first amplification step (target-specific) of the library preparation for Illumina MiSeq sequencing.** Italic lowercase are binding sites for the Illumina sequencing primers. Bold uppercase letters highlight the barcode/index sequence. Italic uppercase letters highlight the linker, which links the barcode with the *Pseudomonas* 16S rRNA gene complementary primer sequence.

| **Forward Primers** | **Sequence (5’ to 3’)** |
| --- | --- |
| **F1** | *acactctttccctacacgacgctcttccgatct* **AATGGT** *CA* ACTTTAAGTTGGGAGGAAGGG |
| **F2** | *acactctttccctacacgacgctcttccgatct* **ATTCTC** *CA* ACTTTAAGTTGGGAGGAAGGG |
| **F3** | *acactctttccctacacgacgctcttccgatct* **ATACCT** *CA* ACTTTAAGTTGGGAGGAAGGG |
| **F4** | *acactctttccctacacgacgctcttccgatct* **AATCCA** *CA* ACTTTAAGTTGGGAGGAAGGG |
| **F5** | *acactctttccctacacgacgctcttccgatct* **ATTGAG** *CA* ACTTTAAGTTGGGAGGAAGGG |
| **F6** | *acactctttccctacacgacgctcttccgatct* **CCTTGA** *CA* ACTTTAAGTTGGGAGGAAGGG |
| **F7** | *acactctttccctacacgacgctcttccgatct* **CCGTAG** *CA* ACTTTAAGTTGGGAGGAAGGG |
| **F8** | *acactctttccctacacgacgctcttccgatct* **CGGAAC** *CA* ACTTTAAGTTGGGAGGAAGGG |
| **F9** | *acactctttccctacacgacgctcttccgatct* **CGATTA** *CA* ACTTTAAGTTGGGAGGAAGGG |
| **F10** | *acactctttccctacacgacgctcttccgatct* **CACATA** *CA* ACTTTAAGTTGGGAGGAAGGG |
| **F11** | *acactctttccctacacgacgctcttccgatct* **GAATCT** *CA* ACTTTAAGTTGGGAGGAAGGG |
| **F12** | *acactctttccctacacgacgctcttccgatct* **GATAAG** *CA* ACTTTAAGTTGGGAGGAAGGG |
| **F13** | *acactctttccctacacgacgctcttccgatct* **GGATGC** *CA* ACTTTAAGTTGGGAGGAAGGG |
| **F14** | *acactctttccctacacgacgctcttccgatct* **GAACGG** *CA* ACTTTAAGTTGGGAGGAAGGG |
| **F15** | *acactctttccctacacgacgctcttccgatct* **GGACTT** *CA* ACTTTAAGTTGGGAGGAAGGG |
| **F16** | *acactctttccctacacgacgctcttccgatct* **TGAGGA** *CA* ACTTTAAGTTGGGAGGAAGGG |
| **F17** | *acactctttccctacacgacgctcttccgatct* **TACCCA** *CA* ACTTTAAGTTGGGAGGAAGGG |
| **F18** | *acactctttccctacacgacgctcttccgatct* **TTCAAC** *CA* ACTTTAAGTTGGGAGGAAGGG |
| **F19** | *acactctttccctacacgacgctcttccgatct* **TCATGT** *CA* ACTTTAAGTTGGGAGGAAGGG |
| **F20** | *acactctttccctacacgacgctcttccgatct* **TCGCTT** *CA* ACTTTAAGTTGGGAGGAAGGG |
| **Reverse Primer** | **Sequence (5’ to 3’)** |
| **R0** | *gtgactggagttcagacgtgtgctcttccgatct* ACACAGGAAATTCCACCACCC |

**Suplementary Table 3B. Nucleotide sequences of primers, targeting the 16S rRNA gene, used in the second amplification step (multiplexing) of the library preparation for Illumina MiSeq Sequencing.** Underlined lowercase letters are binding sites for the Illumina’s flow cell and italic lowercase letters are binding sites for the Illumina sequencing primers. Bold uppercase letters highlight the index sequence.

| **Reverse Primers** | **Sequence (5’ to 3’)** |
| --- | --- |
| **IDX_R1** | caagcagaagacggcatacgagat **CGTGAT** *gtgactggagttcagacgtgtgctcttccgatct* |
| **IDX_R2** | caagcagaagacggcatacgagat **ACATCG** *gtgactggagttcagacgtgtgctcttccgatct* |
| **IDX_R3** | caagcagaagacggcatacgagat **GCCTAA** *gtgactggagttcagacgtgtgctcttccgatct* |
| **IDX_R4** | caagcagaagacggcatacgagat **TGGTCA** *gtgactggagttcagacgtgtgctcttccgatct* |
| **IDX_R5** | caagcagaagacggcatacgagat **CACTGT** *gtgactggagttcagacgtgtgctcttccgatct* |
| **IDX_R6** | caagcagaagacggcatacgagat **ATTGGC** *gtgactggagttcagacgtgtgctcttccgatct* |
| **IDX_R7** | caagcagaagacggcatacgagat **GATCTG** *gtgactggagttcagacgtgtgctcttccgatct* |
| **IDX_R8** | caagcagaagacggcatacgagat **TCAAGT** *gtgactggagttcagacgtgtgctcttccgatct* |
| **IDX_R9** | caagcagaagacggcatacgagat **CTGATC** *gtgactggagttcagacgtgtgctcttccgatct* |
| **IDX_R10** | caagcagaagacggcatacgagat **AAGCTA** *gtgactggagttcagacgtgtgctcttccgatct* |
| **IDX_R11** | caagcagaagacggcatacgagat **GTAGGC** *gtgactggagttcagacgtgtgctcttccgatct* |
| **IDX_R12** | caagcagaagacggcatacgagat **TACAAG** *gtgactggagttcagacgtgtgctcttccgatct* |
| **Forward primer** |  |
| **Illu_Mplex** | aatgatacggcgaccaccgagatct *acactctttccctacacgacgctcttccgatct* |

**Supplementary Table 4. Weighted Spearman rank correlation (r_s_) between replicates coefficient calculated for nine water samples.** Values shown as mean ± SD. *Pseudomonas* template concentration (genome copies): low (<10^2^); medium (10^2^-10^4^); high (>10^4^).

| Sample | Site | *Pseudomonas* concentration | r_s_ |
| --- | --- | --- | --- |
| 1 | cooling tower | low | 0.78 ± 0.05 |
| 2 | cooling tower | low | 0.72 ± 0.08 |
| 3 | cooling tower | low | 0.76 ± 0.05 |
| 4 | cooling tower | medium | 0.78 ± 0.05 |
| 5 | river | medium | 0.81 ± 0.04 |
| 6 | river | medium | 0.76 ± 0.02 |
| 7 | river | high | 0.97 ± 0.01 |
| 8 | river | high | 0.87 ± 0.04 |
| 9 | drinking | low | 0.75 ± 0.05 |

**Supplementary Table 5. Richness and diversity estimators derived from *Pseudomonas* genus 16S rRNA gene sequencing libraries of cooling tower water samples from January 2013 to December 2014.** S_obs_, number of phylotypes observed; *H’*, Shannon diversity index; *E*, Shannon evenness index as percentage.

| Sample | S_obs_ | *H’* | *E* |
| --- | --- | --- | --- |
| January 2013 | 31 | 2.83 | 57.2 |
| February 2013 | 35 | 3.47 | 67.8 |
| March 2013 | 29 | 3.41 | 70.4 |
| April 2013 | 15 | 1.59 | 40.8 |
| May 2013 | 30 | 3.60 | 73.8 |
| June 2013 | 36 | 4.16 | 80.5 |
| July 2013 | 33 | 1.00 | 19.9 |
| August 2013 | 29 | 1.87 | 38.3 |
| September 2013 | 9 | 2.14 | 67.4 |
| October 2013 | 13 | 1.79 | 48.6 |
| November 2013 | 13 | 2.08 | 56.5 |
| December 2013 | 19 | 1.36 | 32.3 |
| January 2014 | 10 | 1.06 | 32.0 |
| February 2014 | 20 | 1.39 | 32.6 |
| March 2014 | 24 | 2.28 | 49.9 |
| April 2014 | 25 | 2.65 | 57.2 |
| May 2014 | 21 | 1.54 | 35.4 |
| June 2014 | 23 | 2.37 | 52.0 |
| July 2014 | 30 | 3.03 | 62.0 |
| August 2014 | 32 | 4.04 | 80.7 |
| September 2014 | 30 | 4.08 | 83.2 |
| October 2014 | 11 | 1.54 | 44.6 |
| November 2014 | 21 | 2.35 | 53.3 |
| December 2014 | 30 | 3.41 | 69.8 |

**Supplementary Table 6. Absolute quantification of *Pseudomonas* spp. and both relative and absolute quantification of *P. aeruginosa*.** *Pseudomonas* cell counts per liter of cooling tower water (Pse_counts_) calculated using qPCR data and TCC determined by epifluorescence microscopy. *P. aeruginosa* relative abundance in the *Pseudomonas* community (Pa_NGS_) determined by the *Pseudomonas* genus-specific NGS Illumina-based assay. *P. aeruginosa* cell counts per liter of cooling tower water(Pa_counts_) calculated as a function of the relative abundances obtained with NGS assay.

| Sample | TCC L^-1^ | Pse_counts_ L^-1^ | Pa_NGS_ (%) | Pa_counts_ |
| --- | --- | --- | --- | --- |
| January 2013 | 1.8 x 10^9^ | 1.7 x 10^4^ | 2.1 | 3.5 x 10^2^ |
| February 2013 | 1.2 x 10^9^ | 1.0 x 10^5^ | 2.8 | 2.9 x 10^3^ |
| March 2013 | 5.1 x 10^8^ | 7.4 x 10^3^ | 9.0 | 6.7 x 10^2^ |
| April 2013 | 9.3 x 10^8^ | 1.3 x 10^4^ | 1.0 | 1.3 x 10^2^ |
| May 2013 | 2.0 x 10^9^ | 1.7 x 10^4^ | 27.4 | 4.6 x 10^3^ |
| June 2013 | 6.1 x 10^8^ | 3.4 x 10^3^ | 7.3 | 2.5 x 10^2^ |
| July 2013 | 9.9 x 10^8^ | 4.3 x 10^4^ | 87.5 | 3.7 x 10^4^ |
| August 2013 | 4.0 x 10^8^ | 2.9 x 10^4^ | 64.7 | 1.9 x 10^4^ |
| September 2013 | 4.8 x 10^8^ | 3.1 x 10^4^ | 26.9 | 8.3 x 10^3^ |
| October 2013 | 1.2 x 10^9^ | 3.3 x 10^4^ | 21.5 | 7.1 x 10^3^ |
| November 2013 | 7.5 x 10^8^ | 1.9 x 10^4^ | 37.9 | 7.2 x 10^3^ |
| December 2013 | 9.7 x 10^8^ | 1.8 x 10^5^ | n.d. | - |
| January 2014 | 6.9 x 10^8^ | 1.3 x 10^5^ | n.d. | - |
| February 2014 | 1.3 x 10^9^ | 4.6 x 10^4^ | 0.1 | 5.8 x 10^1^ |
| March 2014 | 9.0 x 10^8^ | 6.5 x 10^4^ | 0.7 | 4.4 x 10^2^ |
| April 2014 | 7.1 x 10^8^ | 7.3 x 10^3^ | 0.5 | 3.6 x 10^1^ |
| May 2014 | 4.5 x 10^8^ | 7.1 x 10^3^ | 1.6 | 1.1 x 10^2^ |
| June 2014 | 4.3 x 10^8^ | 1.0 x 10^4^ | 1.7 | 1.7 x 10^2^ |
| July 2014 | 6.1 x 10^8^ | 3.5 x 10^4^ | <0.1 | 6.7 x 10^0^ |
| August 2014 | 6.3 x 10^8^ | 1.3 x 10^4^ | 4.0 | 5.3 x 10^2^ |
| September 2014 | 1.2 x 10^9^ | 1.7 x 10^4^ | 4.0 | 6.8 x 10^2^ |
| October 2014 | 8.7 x 10^8^ | 2.0 x 10^5^ | 67.3 | 1.3 x 10^5^ |
| November 2014 | 1.5 x 10^9^ | 1.0 x 10^5^ | 55.7 | 5.8 x 10^4^ |
| December 2014 | 1.6 x 10^9^ | 1.0 x 10^4^ | 21.3 | 2.1 x 10^3^ |
